# Regulating mitochondrial oxidative phosphorylation and MAPK signaling: wedelolactone as a novel therapeutic for radiation-induced thrombocytopenia

**DOI:** 10.3389/fphar.2025.1508215

**Published:** 2025-04-30

**Authors:** Zhichao Li, Qinyao Li, Shuang Wu, Xinyue Mei, Xiao Qi, Sheng Liu, Gan Qiao, Hongping Shen, Jiesi Luo, Jing Zeng, Feihong Huang, Rong Li, Long Wang

**Affiliations:** ^1^ Department of Pharmacology, School of Pharmacy, Southwest Medical University, Luzhou, Sichuan, China; ^2^ School of Basic Medical Sciences, Southwest Medical University, Luzhou, Sichuan, China; ^3^ Clinical Trial Center, The Affiliated Traditional Chinese Medicine Hospital of Southwest Medical University, Luzhou, Sichuan, China; ^4^ Drug Discovery Research Center, Southwest Medical University, Luzhou, Sichuan, China; ^5^ Laboratory for Cardiovascular Pharmacology of Department of Pharmacology, The School of Pharmacy, Southwest Medical University, Luzhou, Sichuan, China

**Keywords:** wedelolactone, thrombocytopenia, oxidative phosphorylation, megakaryocyte differentiation, thrombopoiesis

## Abstract

**Introduction:**

Radiation-induced thrombocytopenia (RIT) is a serious complication of cancer radiotherapy, for which therapeutic options are limited. This study investigates wedelolactone (WED), a metabolite of a botanical drug, as a potential treatment for RIT.

**Methods:**

*In vitro* experiments were conducted using Meg‐01 and K562 cell lines to evaluate the effects of WED on megakaryocyte differentiation and maturation. Flow cytometry and phalloidin staining were employed to assess the expression of megakaryocyte‐specific markers CD41 and CD61, as well as nuclear polyploidization. A mouse model of RIT was established to assess the efficacy of WED in restoring platelet counts and regulating hematopoiesis. RNA sequencing and western blot analyses were performed to explore the underlying molecular mechanisms.

**Results:**

*In vitro* experiments revealed that WED enhanced megakaryocyte differentiation in a dose‐dependent manner, increasing the expression of lineage‐specific markers CD41 and CD61, and promoting polyploidization and cytoskeletal reorganization. *In vivo*, WED significantly restored platelet counts in the mouse model of RIT and promoted the production of hematopoietic stem cells (HSCs), megakaryocytes, and reticulated platelets. RNA sequencing and western blot revealed that WED-induced megakaryocyte differentiation involves the regulation of mitochondrial oxidative phosphorylation mediated by the AMPK signaling pathway and activation of the MAPK signaling pathway. Inhibition of mitochondrial oxidative phosphorylation or MAPK signaling suppressed WED‐induced megakaryocyte differentiation, highlighting the central role of these pathways.

**Discussion:**

These findings indicate that WED could be a promising therapeutic candidate for RIT, acting through the modulation of oxidative phosphorylation and MAPK signaling pathways to enhance thrombopoiesis.

## Graphical Abstract

**Figure:**
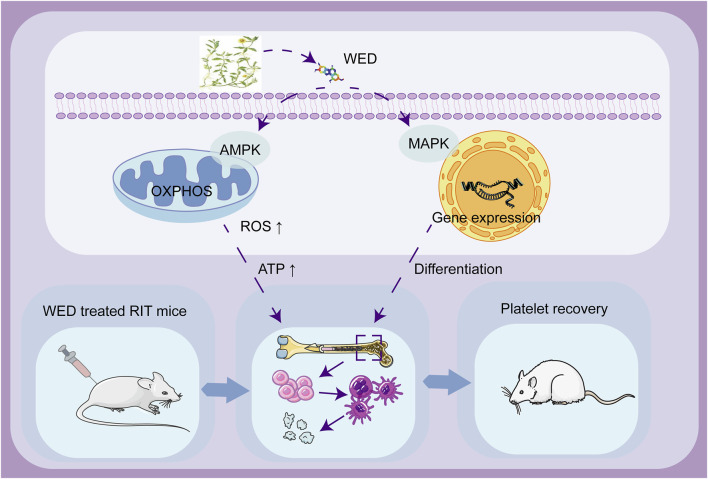

## 1 Introduction

Thrombocytopenia is a prevalent hematological disorder defined by a peripheral blood platelet count below the normal threshold of 150 × 10^9^/L (Raghunathan et al., 2022). It often results in bleeding tendencies, coagulation abnormalities, and, in severe cases, life-threatening conditions (Greenberg, 2017). The etiology of thrombocytopenia is multifactorial, frequently linked to either impaired platelet production or accelerated platelet clearance (Chen et al., 2022). This condition can arise from diverse causes, including genetic predispositions, acquired disorders, microbial infections, or as a consequence of radiotherapy and chemotherapy. Thrombocytopenia significantly worsens patient prognosis and restricts the efficacy of treatments, particularly in those undergoing radiotherapy and chemotherapy (Du et al., 2023). Additionally, it can precipitate serious complications, such as arterial and venous thrombosis, ischemic stroke, and myocardial infarction, thereby contributing substantially to global morbidity and mortality (Xu et al., 2016). Consequently, the rapid and effective elevation of platelet counts is a critical therapeutic objective in the management of thrombocytopenia.

Platelets are small cytoplasmic fragments shed from megakaryocytes in the bone marrow (BM), playing critical roles in several physiological processes, including hemostasis, thrombosis, and immunomodulation. With an average lifespan of 7–10 days, continuous platelet production is essential to maintain normal platelet counts (Liu et al., 2014). In healthy individuals, platelets are produced from hematopoietic stem cells (HSCs) through a complex series of differentiation and maturation processes known as thrombopoiesis. HSCs differentiate into megakaryocytes, which undergo differentiation and polyploidization to form platelet precursors that release platelets into the vascular lumen (Ghalloussi et al., 2019). This process is regulated by several key signaling pathways, such as JAK/STAT, PI3K/AKT, and MAPK. These activated pathways regulate the expression of critical transcription factors, including GATA1, RUNX1, NF-E2, FOS, and TAL1, which are pivotal in megakaryocyte differentiation, maturation, and thrombopoiesis (Yang et al., 2022; Lai et al., 2023). Furthermore, recent studies have suggested that mitochondrial metabolism may represent a novel regulatory mechanism in megakaryocyte differentiation. For instance, atmospheric particulate matter has been reported to induce thrombopoiesis by modulating mitochondrial oxidative phosphorylation (Jin et al., 2021). Similarly, virodhamine has been shown to promote megakaryocyte differentiation by regulating mitochondrial function (Sharma et al., 2021), while *Justicia adhatoda* L. [Acanthaceae] induces differentiation via mitochondrial reactive oxygen species (ROS) production (Gutti et al., 2018). These findings underscore the emerging significance of mitochondrial pathways in regulating megakaryocyte maturation and thrombopoiesis.

The primary treatments for thrombocytopenia currently include pharmacological therapy, platelet transfusion, and dietary modification. Pharmacological interventions typically involve glucocorticoids, immunoglobulins, and thrombopoietin receptor agonists to enhance platelet production. However, these approaches often come with significant drawbacks, including high relapse rates, limited efficacy, and the development of drug tolerance (Lasne et al., 2020). Platelet transfusion, while capable of rapidly increasing platelet levels, is not a sustainable long-term solution due to limited donor resources and the risk of transfusion-related reactions (Katsube et al., 2019). Consequently, significant challenges persist in the management of thrombocytopenia. There is an essential demand for the development of innovative and effective interventions that can safely and rapidly elevate platelet counts and improve patient outcomes.

The metabolites of botanical drugs are widely recognized as valuable precursors for drug development due to their unique chemical structures and diverse biological activities (Sun et al., 2020). The metabolites derived from plants, microorganisms, and marine organisms offer significant chemical diversity, providing novel insights for drug discovery. Wedelolactone (WED), a polyphenol sharing a coumarin skeleton with a benzofuran moiety at C-3 and C-4, belongs to a class of secondary metabolites renowned for diverse pharmacological effects. Isolated from traditional medicinal plants such as *Eclipta prostrata* (L.) L. [Asteraceae], *Sphagneticola calendulacea* (L.) Pruski [Asteraceae], WED has been extensively studied for its potent bioactivities, including antifibrotic, anti-inflammatory, anticancer, and antidiabetic properties (Tu et al., 2021; Swami et al., 2023; Zhang J. et al., 2023). These effects are primarily attributed to its molecular structure, which facilitates interactions with various cellular targets, influencing key signaling pathways (Tu et al., 2021; Zhang J. et al., 2023). The intrinsic molecular properties of WED, such as its stability and reactivity, drive ongoing research in the biomedical and pharmaceutical fields, particularly for the development of novel therapeutic interventions (Swami et al., 2023). In addition, the use of botanical drug metabolites like WED is increasingly recognized as a promising alternative to synthetic drugs, offering a more sustainable and often less toxic approach to treatment. The potential of WED as an effective therapeutic agent underscores the importance of exploring botanical drug metabolites in modern medicine (Swami et al., 2023). WED was previously identified as a potential therapy for thrombocytopenia through virtual screening, demonstrating its ability to restore platelet counts in radiation-induced thrombocytopenia (RIT) mice models without systemic toxicity (Mo et al., 2023). However, the precise mechanism underlying WED’s efficacy in treating thrombocytopenia remains unclear. The molecular mechanism underlying WED-induced megakaryocyte differentiation was investigated using RNA sequencing. Experimental validation further confirmed that WED promotes megakaryocyte differentiation and maturation by regulating mitochondrial metabolism and activating the MAPK pathway. These findings emphasize the potential impact of mitochondrial metabolism in hematopoiesis and indicate that WED could be a prospective candidate for treating RIT clinically.

## 2 Materials and methods

### 2.1 Chemicals

WED, with a purity exceeding 99.66% was ascertained through High Performance Liquid Chromatography, was procured by Chengdu Pusi Biotechnology Co., Ltd. and dissolved as per the instructions.

### 2.2 Cell culture

The human chronic myeloid leukemia cell line (K562) and human megakaryocytic leukemia cell line (Meg-01) were obtained from the American Type Culture Collection (Bethesda, MD, United States). These cells were cultured in RPMI-1640 medium, with 10% fetal bovine serum (FBS) and 1% penicillin/streptomycin.

### 2.3 Quantification of cells surface markers

Following the 5-day treatment involving WED (2.5, 5, and 10 μM) along with Phorbol 12-myristate 13-acetate (PMA) (Mo et al., 2023), the cells were collected and were subsequently incubated with FITC-CD41 and PE-CD61(4A Biotech, Beijing, China) antibodies for a duration of 30 min, shielded from light. The quantification of CD41 and CD61 expression levels were quantified using flow cytometer (BD Biosciences, San Jose, CA, USA).

### 2.4 DNA ploidy analysis

Cells were harvested and washed three times with phosphate-buffered saline (PBS), as previously described. They were then fixed overnight at 4°C, followed by two additional washes with PBS. Next, the cells were incubated with propidium iodide/RNase staining buffer for 15 min at room temperature. Finally, the stained cells were analyzed using flow cytometry.

### 2.5 Phalloidin staining

Cells were collected as previously outlined and fixed with 4% paraformaldehyde for 10 min, followed by permeabilization with 0.5% Triton X-100. They were then shielded from light and stained with tetramethylrhodamine-labeled ghost pen cyclic peptide (Solarbio, Beijing, China) for 1 h. Afterward, the nuclei were counterstained with 100 nM 4′,6-diamidino-2-phenylindole (Solarbio, Beijing, China) for 30 s. Fluorescence images were captured using a fluorescence microscope (Leica, Wetzlar, Germany).

### 2.6 Animals

Kunming (KM) mice, aged 8–10 weeks, were acquired from Liaoning Changsheng Biological Co. Ltd. They were provided a standard diet and kept in an environment with a 12-hour light/dark cycle.

### 2.7 Establishment and treatment of RIT mice model

The mice were divided randomly into four groups: control group, RIT model group, recombinant human thrombopoietin (TPO) positive group (3000 U/kg) (Lin et al., 2021), and WED (2.5, 5, and 10 mg/kg) group (Mo et al., 2023). All groups, except the control group, received total body irradiation (TBI) with 4 Gy X-rays at a dose rate of 4 Gy/min to establish thrombocytopenic model mice. Briefly, KM mice were irradiated in well-ventilated acrylic plastic boxes at room temperature. The boxes were positioned at the center of the irradiation field within the 95% isodose region of the irradiator to ensure uniform dose delivery across multiple samples (Du et al., 2023; Wang et al., 2023). Following irradiation, both the control and model groups received daily intraperitoneal injections of normal saline. The TPO and WED groups received intraperitoneal injections of TPO or WED daily for 12 consecutive days, respectively. Blood samples of 40 µL were collected from the ocular venous plexus (days 0, 3, 7, 10, 12) and analysed using a haematology analyser (Sysmex XT-2000iV, Kobe, Japan).

### 2.8 Flow cytometry analysis of BM

Femur cells of mice were harvested (Liu et al., 2024). Antibody labeling followed standard protocols. For haematopoietic stem progenitor analysis, cells were labelled with FITC-conjugated anti-CD34 and PE-conjugated anti-CD117. For megakaryocyte-erythroid progenitor cell analysis, cells were labelled with FITC-conjugated anti-CD41 and PE-conjugated anti-CD117 For megakaryocyte analysis, cells were labelled with FITC-conjugated anti-CD41 and PE-conjugated anti-CD42d. All antibodies were incubated for 20 min and tested by flow cytometry. All antibodies are from Beijing Sizhenbai Biotechnology Co.

### 2.9 Reticulocyte platelet assay

Whole blood from mice orbits was collected and diluted in Tyrode’s solution. Platelets were labeled with anti-CD61-APC antibody and thiazole orange, then incubated for 20 min in the dark before analysis by flow cytometry.

### 2.10 Immunohistochemical analysis

Mice were euthanized, and the femur and spleen were isolated and fixed in 10% paraformaldehyde. The femur was decalcified in a decalcification solution for over a month. Organs were subsequently embedded in paraffin, sectioned, and stained with antibodies against CD41 (Abmart, Shanghai, China).

### 2.11 Mitochondrial assay

Investigated mitochondrial metabolic function using established methods (Chen et al., 2023). Mitochondrial mass was assessed by staining with Mito-Tracker Green (MTG), while Mitochondrial Membrane Potential (MMP) was evaluated using the Mitochondrial Membrane Potential Assay Kit JC-1. Levels of mitochondrial ROS were measured with the ROS Assay Kit. After staining, cells were analyzed by flow cytometry. Additionally, adenosine triphosphate (ATP) levels were quantified following the manufacturer’s instructions using the ATP Assay Kit. All assay reagents were obtained from Beyotime Biotechnology Technologies Ltd.

### 2.12 RNA sequencing

Briefly, total RNA was extracted using TRIzol (Invitrogen, Carlsbad, CA) following the treatment of cells with WED (10 μM) for 3 days. Libraries were sequenced on the Illumina HiSeq xten/NovaSeq 6,000 platform by Shanghai Metso BioMedical Biotechnology Co. Differentially expressed genes (DEGs) were identified using a fold change (FC) of ≥ 1.5 and a *p-*value of < 0.05. DEGs were then subjected to enrichment analyses. The RNA sequencing data were deposited to Sequence Read Archive of National Center for Biotechnology Information, and the accession number was PRJNA1162465.

### 2.13 Western blot

Briefly, samples were loaded onto 10% sodium dodecyl sulfate polyacrylamide gel electrophoresis followed by polyvinylidene difluoride membrane (BioRad, Hercules, CA). Transfer of membrane was performed. Add primary antibody and incubate overnight, then wash three times with PBST (PBS with 2% Tween-20), the secondary antibody was added and incubated at 37°C for 1 h. Antibodies against RAS (Abmart, T56672S), p-MEK1/2 (CST, 9154S), MEK1/2 (CST, 8727 S), p-ERK1/2 (Abmart, TA1015S), ERK1/2 (Abmart, TA0155), p-AMPK (Abmart, T55608F), AMPK (Abmart, T55326F), NF-E2 (Proteintech, United States, 11089-1-AP), GATA1 (Proteintech, United States, 10917-2-AP), FOS (Proteintech, United States, 66590-1-Ig), GAPDH (Proteintech, United States, 10494-1-AP).

### 2.14 Statistical analysis

The mean ± standard deviation (SD) of at least three independent repeated trials were used to illustrate all calculated data. Statistical analysis was performed using either Student’s t-test or two-way ANOVA to compare the different groups in this study, with a significance level set at *p* < 0.05.

## 3 Results

### 3.1 WED promotes megakaryocyte differentiation and maturation *in vitro*

**FIGURE 1:**
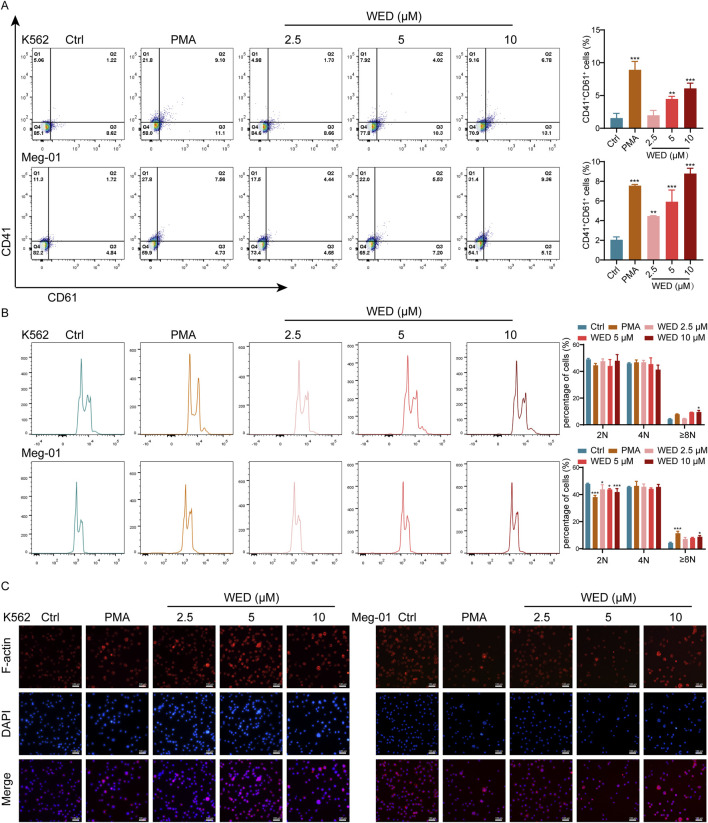
WED induces megakaryocyte differentiation and maturation. **(A)** CD41 and CD61 expression in the cells after 5 days of PMA and WED treatment. The histograms illustrate the proportion of CD41^+^ CD61^+^ cells in each group. **(B)** DNA ploidy analysis of cells using flow cytometry after 5 days of PMA and WED intervention. The histograms display the percentage of 2N, 4N, and ≥ 8N cells. **(C)** Phalloidin staining was conducted on two cell types treated with WED and PMA for 5 days. Scale bar = 100 μm. Data are displayed as mean ± SD (n = 3). *p < 0.05, **p < 0.01, ***p < 0.001 vs. the control group. Ctrl: Control.

Megakaryocyte development involves a process of “excessive hypertrophy”, marked by substantial cell volume expansion, polyploidy formation, and elevated expression of megakaryocyte-specific markers (Tang et al., 2024). To assess whether WED directly influences megakaryocyte differentiation and maturation, we treated Meg-01 and K562 cells with varying doses of WED (2.5, 5, and 10 μM). PMA served as a positive control for *in vitro* megakaryocyte differentiation (Ogura et al., 1988). Flow cytometry analysis, conducted after 5 days of treatment, revealed a dose-dependent increase in the expression of megakaryocyte lineage-specific markers CD41 and CD61 in both WED-treated and PMA-treated groups (Figure 1A).

Polyploidy, a hallmark of mature megakaryocytes (Chase et al., 2010), was also evaluated. Flow cytometry indicated that WED significantly enhanced DNA ploidy (Figure 1B). Given that maturation of megakaryocytes and the formation of proplatelets necessitate the reorganization of the actin cytoskeleton (Machlus et al., 2016), we conducted phalloidin staining. The results indicated that cells in the WED-treated and PMA-treated groups displayed a notable enlargement of cell size and exhibited multilobed, multinucleated nuclei, in contrast to the control group, which showed minimal nuclear maturation (Figure 1C). Together, these findings suggest that WED effectively promotes megakaryocyte differentiation and maturation.

### 3.2 WED promotes platelet recovery in RIT models

**FIGURE 2:**
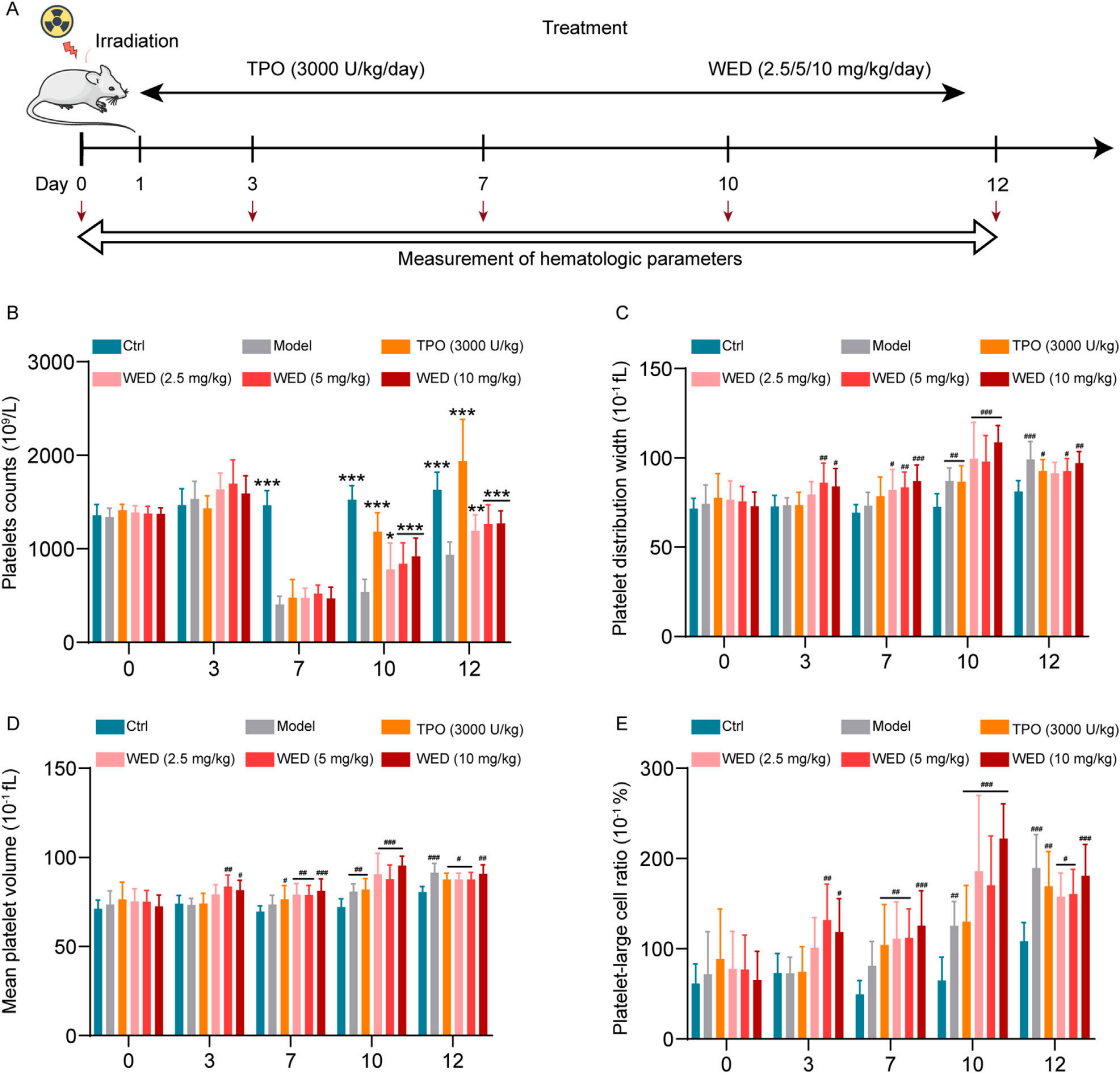
WED promotes platelet recovery in RIT mice. **(A)** Schematic diagram illustrating the study design for the treatment of RIT model mice with WED. **(B–E)** Changes in platelet parameters, including **(B)** platelet counts, **(C)** PDW, **(D)** MPV, **(E)** P-LCR, were measured in peripheral blood on days 0, 3, 7, 10, and 12 after RIT mice were treated with WED. Data are displayed as mean ± SD (n = 10). *p < 0.05, **p < 0.01, ***p < 0.001 vs. the model. ^*#*^ *p* < 0.05, ^*##*^ *p <* 0.01, ^*###*^ *p* < 0.001 vs. control group. Ctrl: Control.

To evaluate the *in vivo* efficacy of WED in treating thrombocytopenia, a RIT mouse model was established using whole-body irradiation with 4 Gy X-rays. Following irradiation, treatment included varying doses of WED (2.5, 5, and 10 mg/kg), with TPO (3000 U/kg) serving as a positive control (Figure 2A). Throughout the experimental period, the health status of the mice was monitored daily. On the seventh day post-irradiation, the mice exhibited lethargy and reduced physical activity. However, by day twelve, no significant differences were observed in body weight, mental state, locomotor activity, or food and water intake among any of the treatment groups. These findings suggest that the mice tolerated WED well at the tested dosage. In irradiated mice, platelet counts declined to their lowest point on day 7, while counts in the control group remained stable. However, from day 7 to day 12, both WED and TPO-treated mice exhibited significant platelet recovery compared to the model group, indicating that WED effectively promotes platelet production in the RIT model (Figure 2B). Platelet distribution width (PDW), mean platelet volume (MPV), and platelet-large cell ratio (P-LCR) were significantly elevated in irradiated mice compared to normal controls (Figures 2C–E). This observation aligns with previous studies showing that surviving megakaryocytes produce larger, more reactive platelets following irradiation, which may serve as an emergency response to compensate for the reduced hemostatic function caused by cytotoxic damage (Du et al., 2023).

### 3.3 WED promotes the production of HSCs, megakaryocytes, and reticulated platelets in RIT mice

**FIGURE 3:**
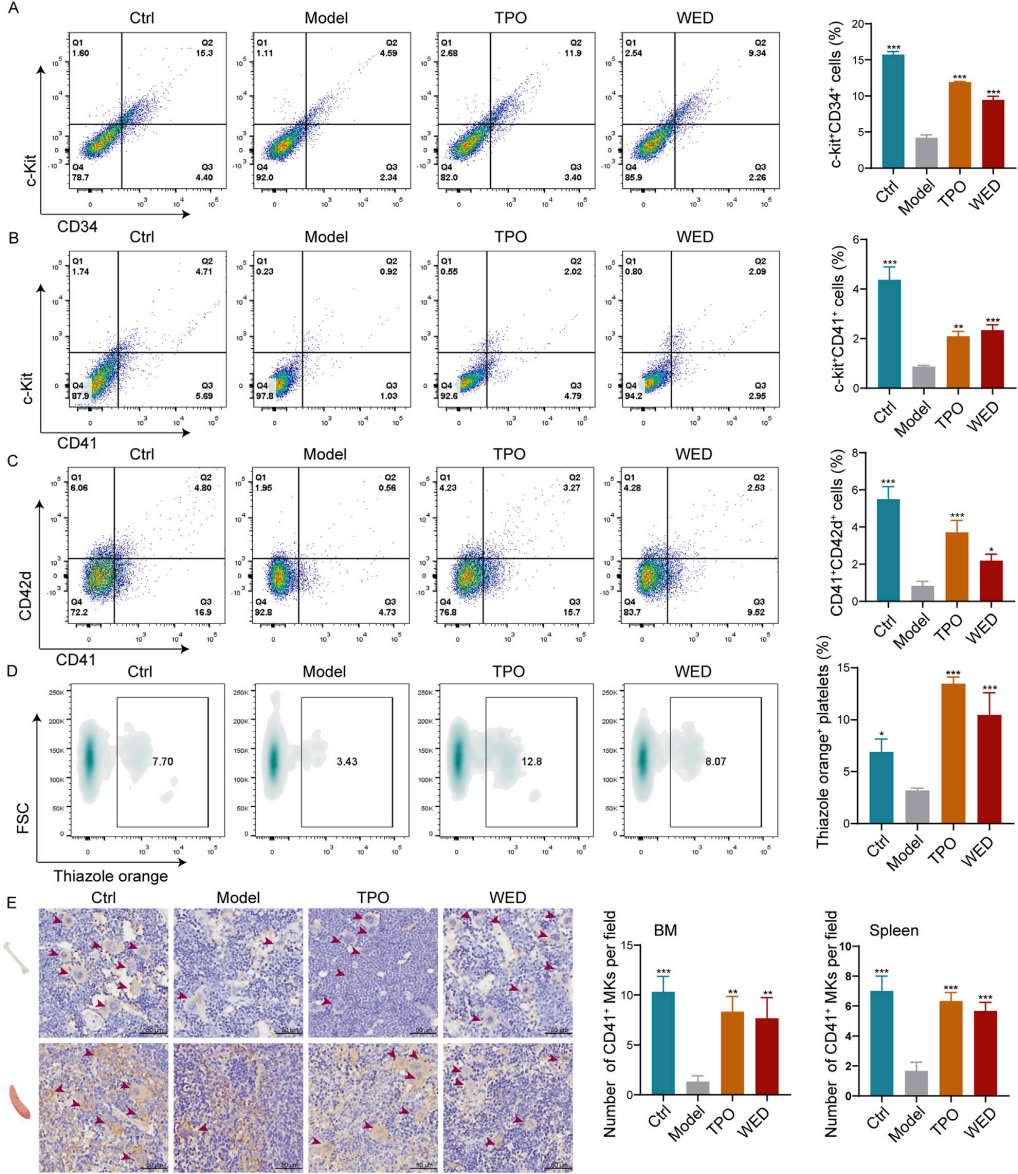
WED stimulates the production of both megakaryocytes and platelets *in vivo*. **(A–C)** Flow cytometry analyses of c-Kit^+^CD34^+^ **(A)**, c-Kit^+^CD41^+^ **(B)**, and CD41^+^CD42d^+^ **(C)** cell percentages in the BM after 10 days of treatment. Histograms represent the proportion of positive cells in each group. **(D)** Thiazole orange-positive platelets in peripheral blood. Histograms represent the proportion of positive platelets in each group. **(E)** Immunohistochemical staining of CD41 in the BM and spleen tissue. The CD41^+^ MKs are indicated by arrows. Scale bar = 50 μm. Histograms represent the proportion of CD41^+^ cells in BM and spleen across groups. Data are displayed as mean ± SD (n = 3). *p < 0.05, **p < 0.01, ***p < 0.001 vs. model group.

It is well established that hematopoietic cells are highly sensitive to radiation and undergo apoptosis immediately following exposure (Li et al., 2016). As the BM is the primary hematopoietic organ (Guo et al., 2021), we examined the percentages of hematopoietic cells in the BM of each group of mice. Flow cytometry analysis revealed significant increases in the percentages of c-Kit^+^CD34^+^ (hematopoietic stem progenitor) (Figure 3A), c-Kit^+^CD41^+^ (megakaryocytic progenitor) (Figure 3B), and CD41^+^CD42d^+^ (megakaryocyte) (Figure 3C) cells in the TPO- and WED-treated groups compared to the model group. These results suggest that WED can promote various stages of megakaryopoiesis.

Previous studies demonstrated a gradual increase in peripheral platelet counts in RIT mice following WED treatment. To investigate the origin of this increase, we used thiazole orange staining to assess the production of new platelets (Kostyak et al., 2014). Flow cytometry analysis showed a significant increase in thiazole orange-positive platelets in both the TPO and WED-treated groups (Figure 3D), indicating enhanced *de novo* platelet production following WED administration. Additionally, as megakaryocytes, predominantly located in the BM and partially in the spleen, are responsible for platelet production (Ma et al., 2019), we conducted immunohistochemical staining for CD41 in the BM and spleen. The data demonstrated a notable increase in megakaryocyte numbers in the BM of TPO- and WED-treated mice compared with model group, with analogous findings in the spleen (Figure 3E). These findings suggest that WED promotes the generation of both megakaryocytes and platelets in RIT mice.

### 3.4 Gene expression profile associated with WED-induced megakaryocyte differentiation

**FIGURE 4:**
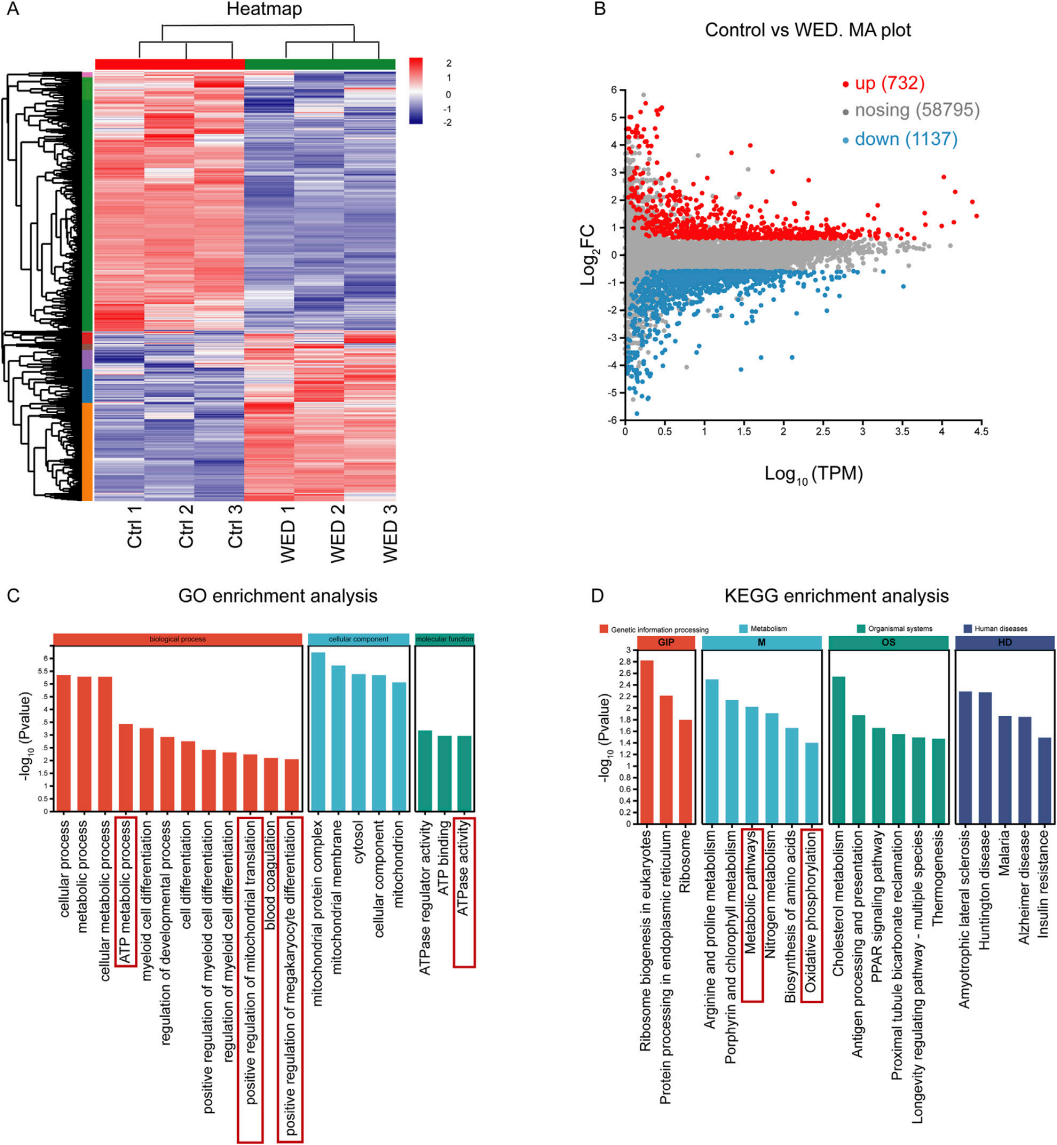
RNA sequencing analysis. **(A)** Heatmap displaying gene expression changes. **(B)** MA plot showing DEGs with upregulated (red) or downregulated (blue). **(C)** GO enrichment analysis. **(D)** KEGG enrichment analysis.

The molecular mechanisms underlying WED-induced megakaryocyte differentiation were investigated through RNA sequencing. A heat map highlighted the changes in overall gene expression between control and WED-treated groups (Figure 4A). Volcano plot revealed 1869 DEGs, with 732 upregulated and 1,137 downregulated in the WED-treated group compared to control (Figure 4B). To further understand the functional roles of the WED-regulated DEGs, GO and KEGG pathway enrichment analyses were conducted. GO analysis revealed that the DEGs were predominantly associated with mitochondrial metabolism-related processes, such as ATP metabolic process, positive regulation of mitochondrial translation, positive regulation of megakaryocyte differentiation, and ATPase activity (Figure 4C). KEGG analysis revealed that these DEGs were significantly enriched in metabolic pathways, oxidative phosphorylation, and related processes (Figure 4D). These findings suggest that mitochondrial metabolism is crucial for WED-induced megakaryocyte differentiation.

### 3.5 WED induces megakaryocyte differentiation by regulating oxidative phosphorylation

**FIGURE 5:**
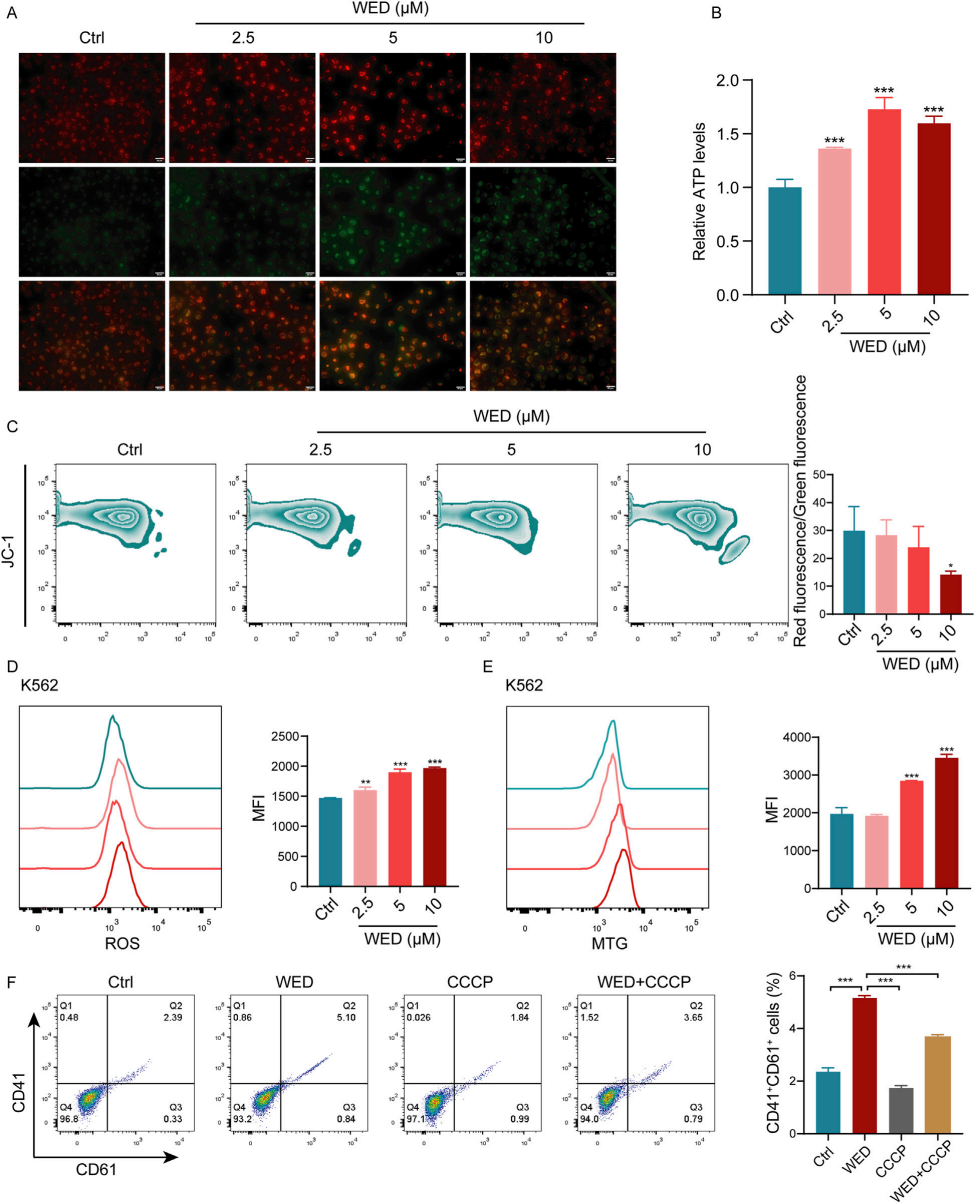
Mitochondrial oxidative phosphorylation contributes to WED-induced megakaryocyte differentiation. **(A)** Fluorescence images of JC-1-stained K562 cells showing changes in MMP. **(B)** Intracellular ATP levels in K562 cells after 5 days of WED treatment. **(C–E)** Flow cytometry evaluation of MMP **(C)**, ROS levels **(D)**, and mitochondrial mass **(E)** in K562 cells after 5 days of WED intervention. *p < 0.05, **p < 0.01, ***p < 0.001. vs the control group. **(F)** Expression of CD41 and CD61. Histograms display the percentage of CD41^+^CD61^+^ cells. Data are displayed as mean ± SD (n = 3). ***p < 0.001 vs. the WED group.

To elucidate the involvement of mitochondrial metabolism and oxidative phosphorylation in WED-induced megakaryocyte differentiation, several mitochondrial metabolism-related indicators were examined, including MMP, cellular ATP levels, ROS content, and mitochondrial mass. The data revealed that WED treatment resulted in a decrease in MMP (Figures 5A,C), accompanied by increased ATP levels (Figure 5B), elevated ROS production (Figure 5D), and enhanced mitochondrial mass (Figure 5E). Analogous results were detected in BM cells of RIT mice (Supplementary Figure S1B,C). The MMP of BM cells increased in both the TPO and WED-treated groups compared to the model group, suggesting that WED may regulate MMP (Supplementary Figure S1A). To further validate the involvement of mitochondrial oxidative phosphorylation in megakaryocyte differentiation, Carbonyl Cyanide m-Chlorophenylhydrazone (CCCP), an inhibitor of mitochondrial oxidative phosphorylation, was added, and flow cytometry analysis showed that CCCP significantly inhibited WED-induced expression of CD41 and CD61 (Figure 5F). Indicating that mitochondrial oxidative phosphorylation is integral to WED-induced megakaryocyte differentiation.

Moreover, RNA sequencing indicated that WED-induced differential genes were significantly enriched in ATP metabolic processes, ATPase regulator activity, ATP binding, ATPase activity, and other pathways related to energy metabolism. Given that AMPK serves as a key regulator of energy metabolism (Herzig and Shaw, 2018), we further investigated the effect of WED on the AMPK signaling pathway. Western blot analysis confirmed that WED significantly promoted AMPK phosphorylation (Supplementary Figure S1D), while the addition of the AMPK inhibitor compound C inhibited WED-induced ATP production (Supplementary Figure S1E). These findings imply that AMPK phosphorylation is essential for WED-regulated mitochondrial oxidative phosphorylation, which may subsequently induce the differentiation of megakaryocytes.

### 3.6 The MAPK signaling pathway is activated during WED-promoted megakaryocyte differentiation

**FIGURE 6:**
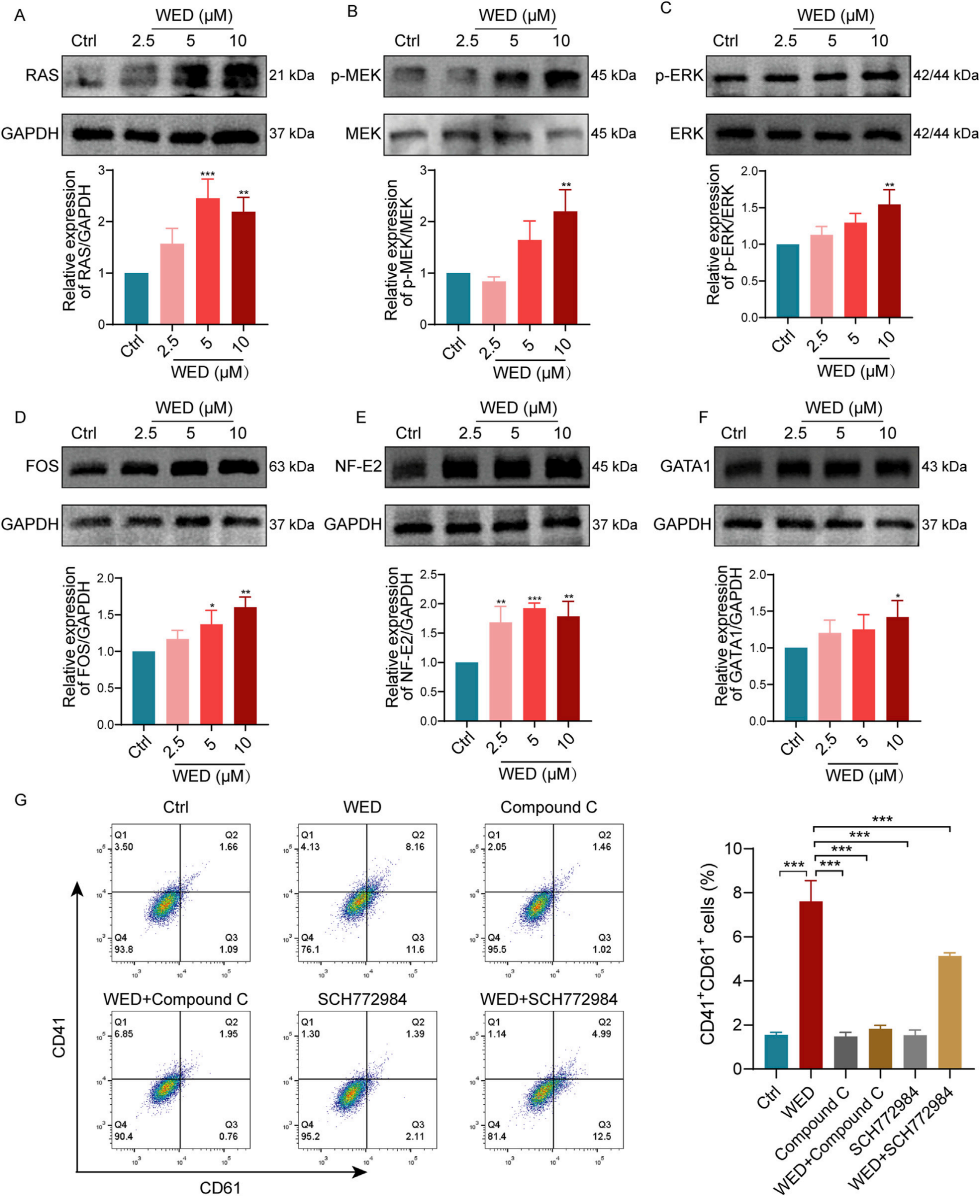
MAPK signaling pathway is necessary for WED-induced megakaryocyte maturation. **(A-F)** Western blot analysis showing the expression levels of RAS, p-MEK, p-ERK, FOS, NF-E2, and GATA1 in cells exposed to WED (2.5, 5, and 10 μM). *p < 0.05, **p < 0.01, ***p < 0.001 vs. the control group. **(G)** Flow cytometry analysis, with histograms displaying the proportion of CD41^+^CD61^+^ cells. Data are displayed as mean ± SD (n = 3). ***p < 0.001 vs. the WED group.

GO analysis revealed that DEGs were enriched in the positive regulation of megakaryocyte differentiation. The MAPK signaling pathway is a crucial intracellular network that governs cellular proliferation and differentiation (Lieu et al., 2017), with numerous studies reporting its role in regulating megakaryocyte differentiation (Manne et al., 2022; Zhou et al., 2024). Additionally, previous research has shown that WED attenuates pulmonary fibrosis in part by activating AMPK and modulating the MAPK signaling pathway (Yang et al., 2019). Therefore, we investigated the role of the MAPK signaling pathway in WED-induced megakaryocyte differentiation. The results show that WED significantly induced the expression of RAS protein and promoted the phosphorylation of MEK and ERK (Figures 6A–C). Additionally, WED enhanced the expression of transcription factors FOS, NF-E2, and GATA1 (Figures 6D–F), which are essential for megakaryocyte differentiation. Flow cytometry analysis further demonstrated that both the AMPK inhibitor compound C and the MAPK inhibitor SCH772984 suppressed WED-induced CD41 and CD61 expressions (Figure 6G). These findings suggest that AMPK-mediated oxidative phosphorylation and the MAPK signaling pathway are essential for WED-induced megakaryocyte differentiation.

## 4 Discussion

Thrombocytopenia is a prevalent and acute complication of chemotherapy and radiotherapy, leading to symptoms such as severe bleeding and increased infection risk, which can interrupt treatment and negatively impact patient outcomes (Zhou et al., 2024). Although current therapeutic strategies like platelet transfusions, thrombopoietin receptor agonists, and glucocorticoids are used to manage thrombocytopenia, their limitations, including side effects and restricted applicability, have driven the search for alternative treatments (Zhang T. et al., 2023). The metabolites of botanical drugs offer a promising avenue for drug development due to their diverse biological activities and low toxicity (Kim et al., 2015). Extensive biomedical research on WED has demonstrated its diverse pharmacological properties, including anticancer, anti-inflammatory, antidiabetic, anti-obesity, antioxidant, antiviral, and anti-aging activities. Additionally, WED exhibits cardiovascular benefits and serine protease inhibition, with notable protective effects on vital organs such as the liver, lungs, bones, and teeth (Cheng et al., 2019; Das et al., 2019; Sarwar et al., 2021; Ha et al., 2023). In this research, we explored the therapeutic potential of WED, a natural bioactive metabolite, in promoting megakaryocyte differentiation and platelet recovery in the context of radiation-induced thrombocytopenia.

The findings of this research indicate that WED significantly promotes the differentiation of megakaryocytes and enhances platelet recovery in the RIT mice model. *In vitro*, WED markedly increased the expression of megakaryocyte lineage markers CD41 and CD61 and promoted megakaryocyte polyploidization, a key step in platelet biogenesis. These findings were further confirmed *in vivo*, where WED-treated mice exhibited significant recovery of platelet counts following irradiation. Flow cytometry analysis revealed that WED enhanced the production of HSCs, megakaryocyte progenitors, mature megakaryocytes, and new platelets in the BM. These results were corroborated by immunohistochemical staining, which showed an elevation in megakaryocyte numbers in both the BM and spleen. Transcriptomic analysis provided insights into the molecular basis of WED’s action, with GO analysis revealed that the DEGs were predominantly associated with mitochondrial metabolism-related processes, such as ATP metabolic process, positive regulation of mitochondrial translation and ATPase activity. Notably, KEGG pathway analyses identified enrichment of oxidative phosphorylation and metabolic processes. To validate the results from RNA sequencing and enhance the reliability of our findings, the molecular mechanism of WED-induced megakaryocyte differentiation was further systematically confirmed using Western blot and flow cytometry.

Mitochondria are essential for cellular energy production through oxidative phosphorylation, which generates ATP, a critical energy source for cell differentiation and proliferation (Valentin-Vega et al., 2012). In this study, WED was found to enhance mitochondrial activity by increasing ATP production and mitochondrial mass while regulating MMP. These changes indicate that WED modulates mitochondrial function to support the energy demands of megakaryocyte differentiation. Furthermore, the addition of CCCP, a mitochondrial oxidative phosphorylation inhibitor, significantly suppressed WED-induced expression of CD41 and CD61, confirming the involvement of mitochondrial oxidative phosphorylation in this process. The process of oxidative phosphorylation leads to the production of ROS (Lu et al., 2022), and increased ROS has been reported to promote megakaryocyte maturation and platelet production (Beaulieu et al., 2011). Additionally, WED activates the AMPK signaling pathway, a key regulator of energy metabolism (Lian et al., 2019). AMPK activation is known to enhance mitochondrial biogenesis and promote oxidative phosphorylation (Weimer et al., 2014; Herzig and Shaw, 2018). Western blot analysis revealed that WED significantly increased the phosphorylation of AMPK, while inhibition of AMPK with compound C attenuated WED-induced ATP production and megakaryocyte differentiation. These results suggest that AMPK-mediated regulation of mitochondrial metabolism is critical for the pro-differentiation effects of WED. The MAPK pathway, particularly the MEK/ERK cascade, is identified as modulating cell proliferation, differentiation, and survival (Wang et al., 2022). Western blot analysis confirmed that WED significantly stimulated the expression of RAS and the phosphorylation of MEK and ERK. Furthermore, WED enhanced the expression of transcription factors FOS, NF-E2, and GATA1, which are vital for the concluding phases of megakaryocyte development and proplatelet synthesis (Kamal et al., 2018; Zhou et al., 2024). Inhibition of the MAPK pathway with SCH772984 suppressed WED-induced CD41/CD61 expression, demonstrating the importance of MAPK signaling in WED-mediated megakaryocyte differentiation.

However, like many polyphenols, WED has been identified as a potential pan-assay interference compound. These compounds are known for their ability to interact with multiple molecular targets and exhibit so-called “promiscuous” binding, which can result in non-specific or artifactual outcomes in various bioassays (Heinrich et al., 2020). To address this limitation, future studies will incorporate additional technical approaches—such as drug target identification techniques, gene knockdown strategies, and multi-omics analyses—to rigorously elucidate the molecular basis of WED’s pro-hematopoietic effects.

**FIGURE 7:**
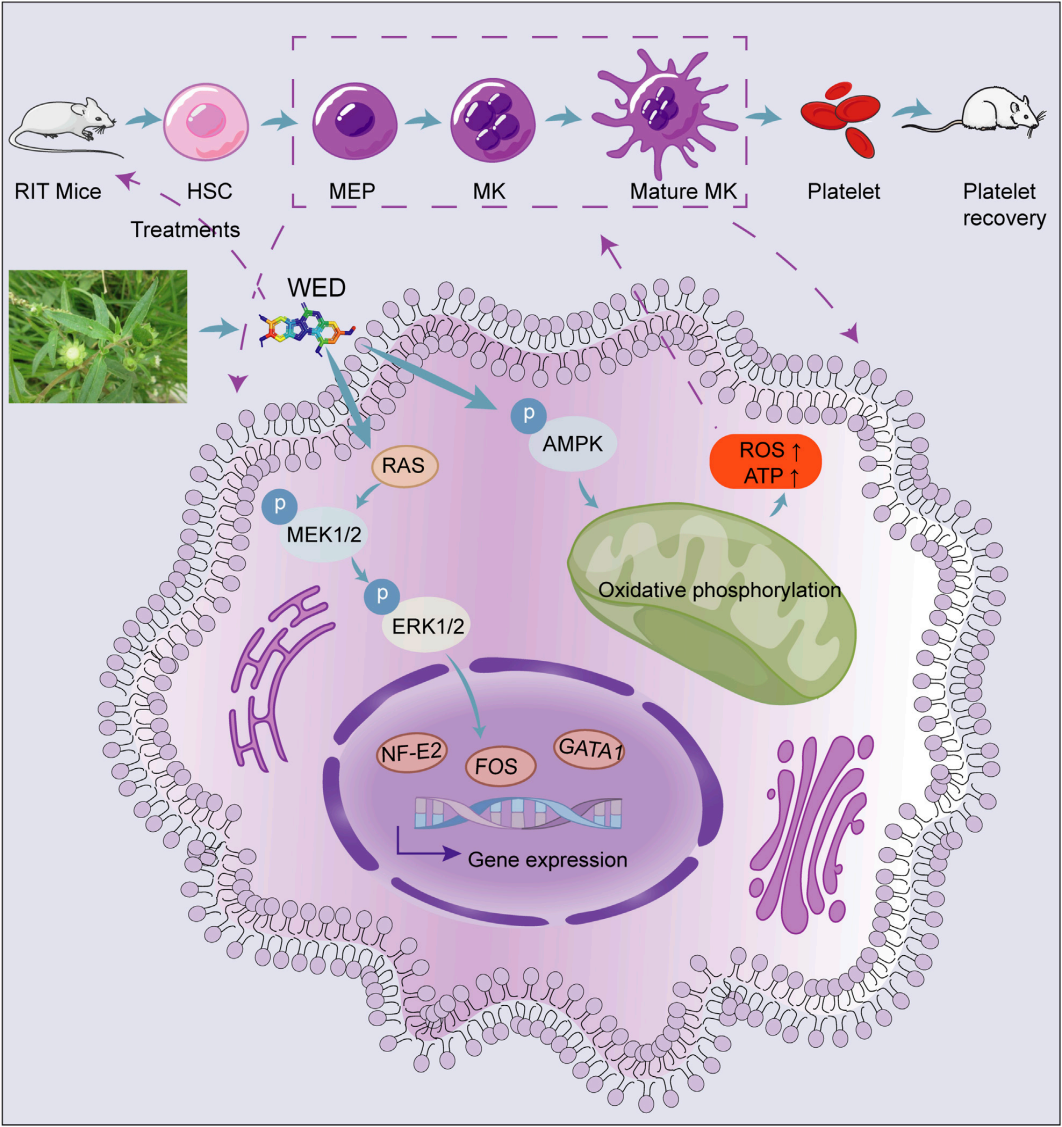
Schematic model showing the role of WED in regulating megakaryocyte differentiation and platelet generation. WED promotes megakaryocyte differentiation and platelet generation by activating the MAPK signaling pathway (RAS/MEK/ERK1/2) and modulating oxidative phosphorylation via the AMPK signaling pathway. Activation of MAPK signaling results in the upregulation of key hematopoietic transcription factors, including FOS, NF-E2, and GATA1, which are essential for megakaryocyte differentiation and maturation. This process ultimately leads to the restoration of platelet counts, as demonstrated in the RIT mice model. MEP: Megakaryocyte-erythroid progenitor.

In summary, this study demonstrates that mitochondrial oxidative phosphorylation and MAPK signaling pathways play a key role in WED-mediated promotion of megakaryocyte differentiation and maturation. Moreover, WED was found to enhance hematopoiesis and restore platelet counts in mice following irradiation-induced injury. Based on both our *in vitro* and *in vivo* findings, WED may represent a promising therapeutic candidate for the treatment of thrombocytopenia. These results provide a theoretical foundation and offer new insights for the development of novel thrombocytopenia therapies (Figure 7).

## 5 Conclusion

These results offer fresh perspectives on the mechanisms by which WED regulates megakaryocyte differentiation and highlight its potential as a therapeutic agent for thrombocytopenia. Future research should explore the clinical applicability of WED and investigate the broader implications of mitochondrial regulation in haematopoietic diseases.

## Data Availability

The datasets presented in this study can be found in online repositories. The names of the repository/repositories and accession number(s) can be found below: https://www.ncbi.nlm.nih.gov/, PRJNA1162465.

## Abbreviations

RIT, Radiation-induced thrombocytopenia; WED, Wedelolactone; HSCs, Hematopoietic stem cells; MK, Megakaryocyte; BM, Bone marrow; K562, Human chronic myeloid leukemia cell line; Meg-01, Human megakaryocytic leukemia cell line; PMA, Phorbol 12-myristate 13-acetate; PBS, Phosphate Buffered Saline; KM, Kunming; TPO, Thrombopoietin; MTG, Mito-Tracker green; ROS, Reactive oxygen species; MMP, Mitochondrial membrane potential; ATP, Adenosine triphosphate; DEGs, Differentially expressed genes; PBST, PBS with 2% Tween-20; PDW, Platelet distribution width; MPV, Mean platelet volume; P-LCR, platelet-large cell ratio; KEGG, Kyoto encyclopedia of genes and genomes; GO, Gene ontology; CCCP, Carbonyl cyanide m-chlorophenylhydrazone.

## References

[B1] Beaulieu L. M. Lin E. Morin K. M. Tanriverdi K. Freedman J. E. (2011). Regulatory effects of TLR2 on megakaryocytic cell function. Blood 117 (22), 5963–5974. 10.1182/blood-2010-09-304949 21454454 PMC3112041

[B2] Chase T. H. Lyons B. L. Bronson R. T. Foreman O. Donahue L. R. Burzenski L. M. (2010). The mouse mutation “thrombocytopenia and cardiomyopathy” (trac) disrupts Abcg5: a spontaneous single gene model for human hereditary phytosterolemia/sitosterolemia. Blood 115 (6), 1267–1276. 10.1182/blood-2009-05-219808 19846887 PMC2826237

[B3] Chen S. Sun K. Xu B. Han S. Wang S. Xu Y. (2023). Akt-mediated mitochondrial metabolism regulates proplatelet formation and platelet shedding post vasopressin exposure. J. Thromb. Haemost. 21 (2), 344–358. 10.1016/j.jtha.2022.11.018 36700501

[B4] Chen Y. Luo L. Zheng Y. Zheng Q. Zhang N. Gan D. (2022). Association of platelet desialylation and circulating follicular helper T cells in patients with thrombocytopenia. Front. Immunol. 13, 810620. 10.3389/fimmu.2022.810620 35450072 PMC9016750

[B5] Cheng M. Lin J. Li C. Zhao W. Yang H. Lv L. (2019). Wedelolactone suppresses IL-1β maturation and neutrophil infiltration in Aspergillus fumigatus keratitis. Int. Immunopharmacol. 73, 17–22. 10.1016/j.intimp.2019.04.050 31078922

[B6] Das S. Mukherjee P. Chatterjee R. Jamal Z. Chatterji U. (2019). Enhancing chemosensitivity of breast cancer stem cells by downregulating SOX2 and ABCG2 using wedelolactone-encapsulated nanoparticles. Mol. Cancer Ther. 18 (3), 680–692. 10.1158/1535-7163.Mct-18-0409 30587555

[B7] Du C. H. Wu Y. D. Yang K. Liao W. N. Ran L. Liu C. N. (2023). Apoptosis-resistant megakaryocytes produce large and hyperreactive platelets in response to radiation injury. Mil. Med. Res. 10 (1), 66. 10.1186/s40779-023-00499-z 38111039 PMC10729570

[B8] Ghalloussi D. Dhenge A. Bergmeier W. (2019). New insights into cytoskeletal remodeling during platelet production. J. Thromb. Haemost. 17 (9), 1430–1439. 10.1111/jth.14544 31220402 PMC6760864

[B9] Greenberg E. M. (2017). Thrombocytopenia: a destruction of platelets. J. Infus. Nurs. 40 (1), 41–50. 10.1097/nan.0000000000000204 28030481

[B10] Guo H. Chang Y. J. Hong Y. Xu L. P. Wang Y. Zhang X. H. (2021). Dynamic immune profiling identifies the stronger graft-versus-leukemia (GVL) effects with haploidentical allografts compared to HLA-matched stem cell transplantation. Cell Mol. Immunol. 18 (5), 1172–1185. 10.1038/s41423-020-00597-1 33408344 PMC8093297

[B11] Gutti U. Komati J. K. Kotipalli A. Saladi R. G. V. Gutti R. K. (2018). Justicia adhatoda induces megakaryocyte differentiation through mitochondrial ROS generation. Phytomedicine 43, 135–139. 10.1016/j.phymed.2018.04.038 29747746

[B12] Ha N. M. Hop N. Q. Son N. T. (2023). Wedelolactone: a molecule of interests. Fitoterapia 164, 105355. 10.1016/j.fitote.2022.105355 36410612

[B13] Heinrich M. Appendino G. Efferth T. Fürst R. Izzo A. A. Kayser O. (2020). Best practice in research - overcoming common challenges in phytopharmacological research. J. Ethnopharmacol. 246, 112230. 10.1016/j.jep.2019.112230 31526860

[B14] Herzig S. Shaw R. J. (2018). AMPK: guardian of metabolism and mitochondrial homeostasis. Nat. Rev. Mol. Cell Biol. 19 (2), 121–135. 10.1038/nrm.2017.95 28974774 PMC5780224

[B15] Jin X. Yu H. Wang B. Sun Z. Zhang Z. Liu Q. S. (2021). Airborne particulate matters induce thrombopoiesis from megakaryocytes through regulating mitochondrial oxidative phosphorylation. Part Fibre Toxicol. 18 (1), 19. 10.1186/s12989-021-00411-4 33985555 PMC8117637

[B16] Kamal T. Green T. N. Hearn J. I. Josefsson E. C. Morel-Kopp M. C. Ward C. M. (2018). N-methyl-d-aspartate receptor mediated calcium influx supports *in vitro* differentiation of normal mouse megakaryocytes but proliferation of leukemic cell lines. Res. Pract. Thromb. Haemost. 2 (1), 125–138. 10.1002/rth2.12068 30046713 PMC5974914

[B17] Katsube T. Shimizu R. Fukuhara T. Kano T. Wajima T. (2019). Pharmacokinetic/pharmacodynamic modelling and simulation of lusutrombopag, a novel thrombopoietin receptor agonist, for the treatment of thrombocytopenia in patients with chronic liver disease undergoing invasive procedures. Clin. Pharmacokinet. 58 (11), 1469–1482. 10.1007/s40262-019-00770-4 31055790 PMC6856258

[B18] Kim N. H. Pham N. B. Quinn R. J. Shim J. S. Cho H. Cho S. M. (2015). The small molecule R-(-)-β-O-methylsynephrine binds to nucleoporin 153 kDa and inhibits angiogenesis. Int. J. Biol. Sci. 11 (9), 1088–1099. 10.7150/ijbs.10603 26221075 PMC4515819

[B19] Kostyak J. C. Bhavanasi D. Liverani E. McKenzie S. E. Kunapuli S. P. (2014). Protein kinase C δ deficiency enhances megakaryopoiesis and recovery from thrombocytopenia. Arterioscler. Thromb. Vasc. Biol. 34 (12), 2579–2585. 10.1161/atvbaha.114.304492 25359855 PMC4239172

[B20] Lai J. Li Y. Ran M. Huang Q. Huang F. Zhu L. (2023). Xanthotoxin, a novel inducer of platelet formation, promotes thrombocytopoiesis via IL-1R1 and MEK/ERK signaling. Biomed. Pharmacother. 163, 114811. 10.1016/j.biopha.2023.114811 37156117

[B21] Lasne D. Pascreau T. Darame S. Bourrienne M. C. Tournoux P. Philippe A. (2020). Measuring beta-galactose exposure on platelets: standardization and healthy reference values. Res. Pract. Thromb. Haemost. 4 (5), 813–822. 10.1002/rth2.12369 33134771 PMC7586713

[B22] Li X. H. Ha C. T. Xiao M. (2016). MicroRNA-30 inhibits antiapoptotic factor Mcl-1 in mouse and human hematopoietic cells after radiation exposure. Apoptosis 21 (6), 708–720. 10.1007/s10495-016-1238-1 27032651 PMC4853469

[B23] Lian X. Wu X. Li Z. Zhang Y. Song K. Cai G. (2019). The combination of metformin and 2-deoxyglucose significantly inhibits cyst formation in miniature pigs with polycystic kidney disease. Br. J. Pharmacol. 176 (5), 711–724. 10.1111/bph.14558 30515768 PMC6365356

[B24] Lieu C. H. Hidalgo M. Berlin J. D. Ko A. H. Cervantes A. LoRusso P. (2017). A phase ib dose-escalation study of the safety, tolerability, and pharmacokinetics of cobimetinib and duligotuzumab in patients with previously treated locally advanced or metastatic cancers with mutant KRAS. Oncologist 22 (9), 1024–1e89. 10.1634/theoncologist.2017-0175 28592615 PMC5599193

[B25] Lin J. Zeng J. Liu S. Shen X. Jiang N. Wu Y. S. (2021). DMAG, a novel countermeasure for the treatment of thrombocytopenia. Mol. Med. 27 (1), 149. 10.1186/s10020-021-00404-1 34837956 PMC8626956

[B26] Liu X. Lai J. Zhang X. Wu A. Zhou L. Li Y. (2024). Harmine promotes megakaryocyte differentiation and thrombopoiesis by activating the Rac1/Cdc42/JNK pathway through a potential target of 5-HTR2A. Phytother. Res. 38, 5134–5149. 10.1002/ptr.8317 39152726

[B27] Liu Z. J. Hoffmeister K. M. Hu Z. Mager D. E. Ait-Oudhia S. Debrincat M. A. (2014). Expansion of the neonatal platelet mass is achieved via an extension of platelet lifespan. Blood 123 (22), 3381–3389. 10.1182/blood-2013-06-508200 24599546 PMC4041172

[B28] Lu C. Yang D. Klement J. D. Colson Y. L. Oberlies N. H. Pearce C. J. (2022). H3K9me3 represses G6PD expression to suppress the pentose phosphate pathway and ROS production to promote human mesothelioma growth. Oncogene 41 (18), 2651–2662. 10.1038/s41388-022-02283-0 35351997 PMC9058223

[B29] Ma Q. Zhu C. Zhang W. Ta N. Zhang R. Liu L. (2019). Mitochondrial PIP3-binding protein FUNDC2 supports platelet survival via AKT signaling pathway. Cell Death Differ. 26 (2), 321–331. 10.1038/s41418-018-0121-8 29786068 PMC6329745

[B30] Machlus K. R. Wu S. K. Stumpo D. J. Soussou T. S. Paul D. S. Campbell R. A. (2016). Synthesis and dephosphorylation of MARCKS in the late stages of megakaryocyte maturation drive proplatelet formation. Blood 127 (11), 1468–1480. 10.1182/blood-2015-08-663146 26744461 PMC4797023

[B31] Manne B. K. Campbell R. A. Bhatlekar S. Ajanel A. Denorme F. Portier I. (2022). MAPK-interacting kinase 1 regulates platelet production, activation, and thrombosis. Blood 140 (23), 2477–2489. 10.1182/blood.2022015568 35930749 PMC9918849

[B32] Mo Q. Zhang T. Wu J. Wang L. Luo J. (2023). Identification of thrombopoiesis inducer based on a hybrid deep neural network model. Thromb. Res. 226, 36–50. 10.1016/j.thromres.2023.04.011 37119555

[B33] Ogura M. Morishima Y. Okumura M. Hotta T. Takamoto S. Ohno R. (1988). Functional and morphological differentiation induction of a human megakaryoblastic leukemia cell line (MEG-01s) by phorbol diesters. Blood 72 (1), 49–60. 10.1182/blood.v72.1.49.bloodjournal72149 2455575

[B34] Raghunathan S. Rayes J. Sen Gupta A. (2022). Platelet-inspired nanomedicine in hemostasis thrombosis and thromboinflammation. J. Thromb. Haemost. 20 (7), 1535–1549. 10.1111/jth.15734 35435322 PMC9323419

[B35] Sarwar S. Alamro A. A. Alghamdi A. A. Naeem K. Ullah S. Arif M. (2021). Enhanced accumulation of cisplatin in ovarian cancer cells from combination with wedelolactone and resulting inhibition of multiple epigenetic drivers. Drug Des. Devel Ther. 15, 2211–2227. 10.2147/dddt.S288707 PMC8164677 34079223

[B36] Sharma D. S. Raghuwanshi S. Kovuru N. Dahariya S. Gautam D. K. Paddibhatla I. (2021). Virodhamine, an endocannabinoid, induces megakaryocyte differentiation by regulating MAPK activity and function of mitochondria. J. Cell Physiol. 236 (2), 1445–1453. 10.1002/jcp.29949 32696508

[B37] Sun G. Hu C. Mei Q. Luo M. Chen X. Li Z. (2020). Uncovering the cytochrome P450-catalyzed methylenedioxy bridge formation in streptovaricins biosynthesis. Nat. Commun. 11 (1), 4501. 10.1038/s41467-020-18336-5 32908132 PMC7481197

[B38] Swami R. K. Nimker S. Narula A. Farooqi H. (2023). Enhanced wedelolactone content in *in vitro*-raised genetically uniform Wedelia chinensis under the influence of CuSO(4). Front. Plant Sci. 14, 1281445. 10.3389/fpls.2023.1281445 38169740 PMC10758438

[B39] Tang X. Liao R. Zhou L. Yi T. Ran M. Luo J. (2024). Genistin: a novel estrogen analogue targeting ERβ to alleviate thrombocytopenia. Int. J. Biol. Sci. 20 (6), 2236–2260. 10.7150/ijbs.90483 38617546 PMC11008259

[B40] Tu Y. Yang Y. Li Y. He C. (2021). Naturally occurring coumestans from plants, their biological activities and therapeutic effects on human diseases. Pharmacol. Res. 169, 105615. 10.1016/j.phrs.2021.105615 33872808

[B41] Valentin-Vega Y. A. Maclean K. H. Tait-Mulder J. Milasta S. Steeves M. Dorsey F. C. (2012). Mitochondrial dysfunction in ataxia-telangiectasia. Blood 119 (6), 1490–1500. 10.1182/blood-2011-08-373639 22144182 PMC3286212

[B42] Wang L. Liu S. Luo J. Mo Q. Ran M. Zhang T. (2023). Targeting a thrombopoietin-independent strategy in the discovery of a novel inducer of megakaryocytopoiesis, DMAG, for the treatment of thrombocytopenia. Haematologica 108 (5), 1394–1411. 10.3324/haematol.2022.282209 36546424 PMC10153531

[B43] Wang Y. Zhang Y. Mi J. Jiang C. Wang Q. Li X. (2022). ANKFN1 plays both protumorigenic and metastatic roles in hepatocellular carcinoma. Oncogene 41 (29), 3680–3693. 10.1038/s41388-022-02380-0 35725908 PMC9287179

[B44] Weimer S. Priebs J. Kuhlow D. Groth M. Priebe S. Mansfeld J. (2014). D-Glucosamine supplementation extends life span of nematodes and of ageing mice. Nat. Commun. 5, 3563. 10.1038/ncomms4563 24714520 PMC3988823

[B45] Xu X. R. Zhang D. Oswald B. E. Carrim N. Wang X. Hou Y. (2016). Platelets are versatile cells: new discoveries in hemostasis, thrombosis, immune responses, tumor metastasis and beyond. Crit. Rev. Clin. Lab. Sci. 53 (6), 409–430. 10.1080/10408363.2016.1200008 27282765

[B46] Yang J. Y. Tao L. J. Liu B. You X. Y. Zhang C. F. Xie H. F. (2019). Wedelolactone attenuates pulmonary fibrosis partly through activating AMPK and regulating raf-MAPKs signaling pathway. Front. Pharmacol. 10, 151. 10.3389/fphar.2019.00151 30890932 PMC6411994

[B47] Yang S. Tang X. Wang L. Ni C. Wu Y. Zhou L. (2022). Targeting TLR2/rac1/cdc42/JNK pathway to reveal that ruxolitinib promotes thrombocytopoiesis. Int. J. Mol. Sci. 23 (24), 16137. 10.3390/ijms232416137 36555781 PMC9787584

[B48] Zhang J. Zhang M. Huo X. K. Ning J. Yu Z. L. Morisseau C. (2023a). Macrophage inactivation by small molecule wedelolactone via targeting sEH for the treatment of LPS-induced acute lung injury. ACS Cent. Sci. 9 (3), 440–456. 10.1021/acscentsci.2c01424 36968547 PMC10037491

[B49] Zhang T. Mo Q. Jiang N. Wu Y. Yang X. Chen W. (2023b). The combination of machine learning and transcriptomics reveals a novel megakaryopoiesis inducer, MO-A, that promotes thrombopoiesis by activating FGF1/FGFR1/PI3K/Akt/NF-κB signaling. Eur. J. Pharmacol. 944, 175604. 10.1016/j.ejphar.2023.175604 36804544

[B50] Zhou L. Ni C. Liao R. Tang X. Yi T. Ran M. (2024). Activating SRC/MAPK signaling via 5-HT1A receptor contributes to the effect of vilazodone on improving thrombocytopenia. Elife 13. 10.7554/eLife.94765 PMC10994662 38573820

