# Supplementary material for: Regulating mitochondrial oxidative phosphorylation and MAPK signaling: wedelolactone as a novel therapeutic for radiation-induced thrombocytopenia
Source: Front Pharmacol. 2025 Apr 30;16:1508215. doi: 10.3389/fphar.2025.1508215 (PMC12075257; doi:10.3389/fphar.2025.1508215)
Supplement: Supplementary file 1 [file DataSheet1.docx]

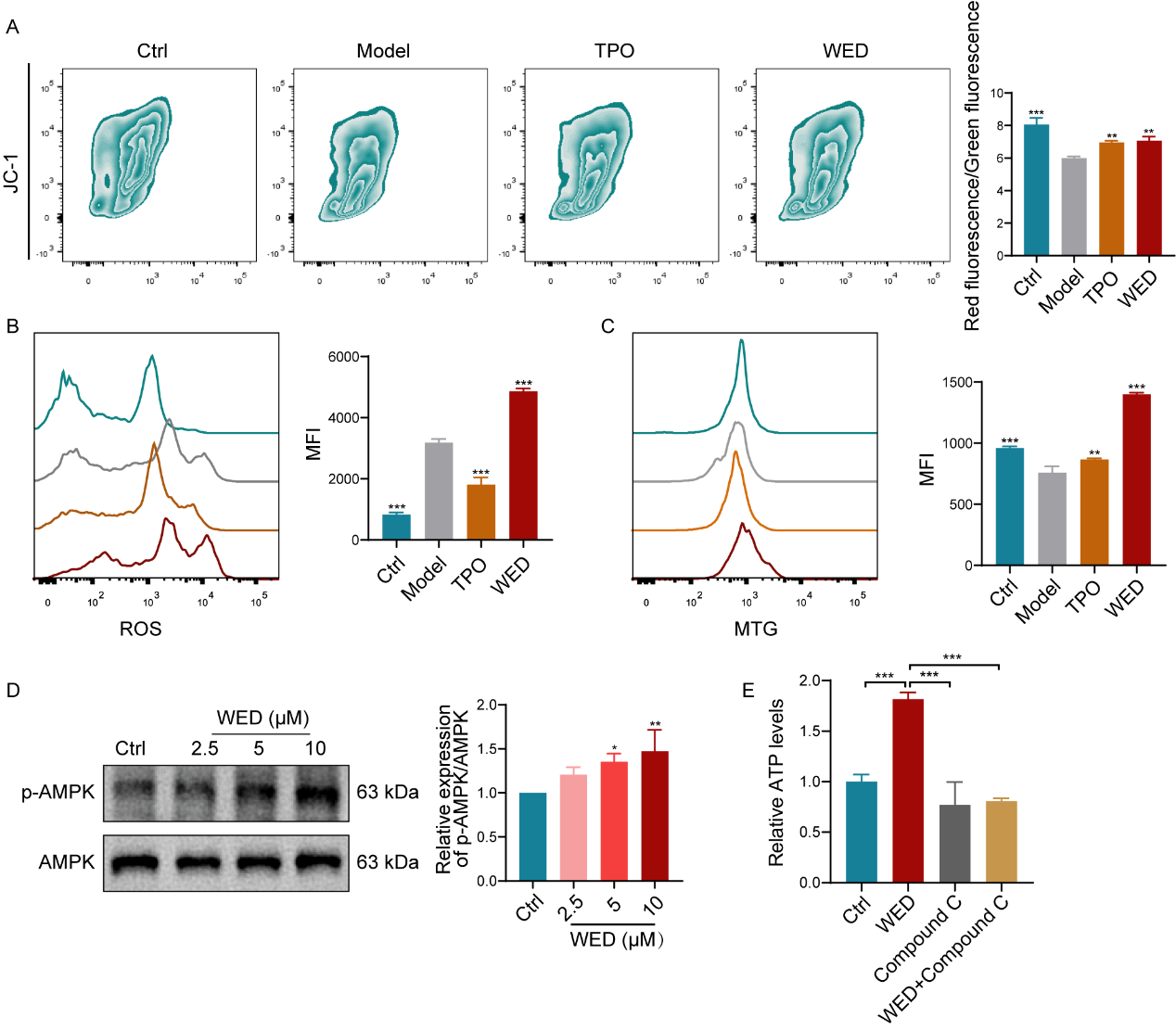
**Supplementary Figure S1**. WED modulates mitochondrial metabolism in BM cells of RIT mice. (A-C) Flow cytometry analysis of mitochondrial membrane potential (A), ROS levels (B), and mitochondrial mass (C) BM cells from mice in each group after 10 days of WED treatment. ***p <* 0.01*, ***p* < 0.001. vs the model group. (D) Western blot analysis of AMPK phosphorylation in K562 cells after five days of WED treatment, with histograms indicating protein expression levels. **p* < 0.05*, **p* < 0.01. vs the control group. (E) Intracellular ATP levels in K562 cells following five days of WED treatment. Data are displayed as mean ± SD (n = 3). ****p* < 0.001. vs the WED group. Ctrl: Control.
